# Association between preoperative serum C-reactive protein level and leukocyte count and postoperative pain after otolaryngological surgery

**DOI:** 10.1007/s00405-023-07980-4

**Published:** 2023-04-20

**Authors:** Katharina Geißler, Robin Brock, Winfried Meißner, Michael Kiehntopf, Orlando Guntinas-Lichius

**Affiliations:** 1 Department of Otorhinolaryngology, Jena University Hospital, Am Klinikum 1, 07747 Jena, Germany; 2 Department of Anesthesiology and Intensive Care Medicine, Jena University Hospital, Jena, Germany; 3 Institute of Clinical Chemistry and Laboratory Diagnostics, Jena University Hospital, Jena, Germany

**Keywords:** C-reactive protein, Leukocyte, Postoperative pain, Otorhinolaryngology

## Abstract

**Purpose:**

To determine whether the preoperative inflammatory serum C-reactive protein (CRP) and leukocyte count (LEUK) are associated with postoperative pain and complaints after otolaryngological surgery.

**Methods:**

Retrospective evaluation of 680 patients (33% female, median age 50 years) receiving otolaryngological surgery between November 2008 and March 2017 in a tertiary university hospital. Postoperative pain on the first postoperative day was assessed using the validated questionnaire of the German-wide project Quality Improvement in Postoperative Pain Treatment (QUIPS) including a numeric rating scale for assessment of postoperative pain (NRS, 0–10). The influence of preoperative parameters including CRP and LEUK on patients' postoperative pain was estimated.

**Results:**

Mean CRP value was 15.6 ± 34.6 mg/l and mean LEUK value 7.8 ± 3.2 Gpt/l. Patients with pharyngeal surgery had the highest CRP values (34.6 ± 52.9 mg/l), highest LEUK values (9.2 ± 4.2 Gpt/l) and the highest pain levels (3.1 ± 2.4 NRS) compared to all other surgical procedures (all *p* < 0.05). Higher postoperative pain was associated with LEUK values > 11.3 Gpt/l (*r* = 0.093, *p* = 0.016) and higher preoperative chronic pain (*r* = 0.127, *p* = 0.001). Multivariate analysis confirmed younger age, female gender, duration of surgery, preoperative chronic pain, type of surgery, and higher LEUK values > 11.3 as independent factors for postoperative pain. Perioperative antibiotics had no effect on the postoperative pain.

**Conclusion:**

Beyond known factors, preoperative LEUK as inflammation marker is an independent predictor for pain on the first postoperative day.

**Supplementary Information:**

The online version contains supplementary material available at 10.1007/s00405-023-07980-4.

## Introduction

Most otorhinolaryngological inflammatory diseases as well as inflammation related to head and neck cancer are associated with acute or even chronic pain. Inflammation could be detected by local symptoms such as swelling, skin and mucosal redness, higher temperature or systemic symptoms like low blood pressure, high body temperature or high levels of inflammation parameters like C-reactive protein (CRP) or leukocytes (LEUK) in blood [1]. Patients with head and neck cancer show positive correlation of CRP level and pain intensity [2]. Patients with chronic pain, mostly due to non-malignant diseases, show a correlation between increased plasma concentration of pro-inflammatory cytokines such as Interleukin-1 (IL-1), IL-2 or IL-6 and increased pain intensity on the numerical rating scale (NRS) [3]. High CRP level was also identified as a risk factor for the occurrence of chronic pain after intensive care treatment [4]. After major laparoscopic abdominal surgery an increase of postoperative CRP level was significantly associated with increase morphine equivalent consumption, whereas preoperative CRP levels were not significantly associated with morphine equivalent consumption [5].

Use of perioperative antibiotics might be related with more postoperative pain as an indirect indication for a correlation between inflammation and postoperative pain [6]. Therefore, the question is if there is an association between the preoperative inflammatory status and postoperative pain level. In the current work, we would like to take up this question and investigate whether the preoperative inflammatory values CRP level and LEUK count affect postoperative pain.

## Methods

### Study design and patients

In this retrospective study, 680 patients who underwent surgery as part of inpatient treatment between November 2008 and March 2017 in the Department of Department of Otorhinolaryngology, Jena University Hospital, Germany, were included. Data was collected by the QUIPS benchmarking project, which was used in previous prospective studies by the Department of Otorhinolaryngology, Jena University Hospital, Germany [7–12]. In addition to these data, the associated preoperative inflammatory parameters in the form of CRP and LEUK were determined for each patient. In most patients, the blood sample was taken the day before surgery. In a minority, the blood sample was taken at the day of surgery. If two samples were available, the value of day nearest to the day of surgery was taken. A positive ethical vote for the retrospective data evaluation was obtained by the Ethics Committee of the Jena University Hospital, Germany. Patients provided informed written consent to have data from their medical records used in research.

### Measurement of postoperative pain and pain-related parameters

The QUIPS questionnaires are presented in detail elsewhere [13]. The QUIPS questionnaires consisted of two parts for each patient: The first part was covering the patient-reported outcome (PRO) parameters of the questionnaire, whereas the second part was filled by the investigator and covered the relevant demographic and clinical parameters like age, gender, type of surgery, anaesthesia and pain management. QUIPS used 11-point numeric rating scales (NRS) to estimate the patient’s pain in activity, maximal pain and minimal pain (0 = no pain; 10 = most imaginable pain). In this study, only the results related to pain in activity are presented. Results related to maximal pain and minimal pains as well as other pain-related parameters were equivalent (data not shown). The first part of the PRO also asks for chronic pain, i.e. pain lasting > 3 months (yes/no). In case of chronic pain, the patient was asked to specify the severity on a NRS (0 = no pain; 10 = most imaginable pain). All patients indicating chronic pain with NRS ≥ 3 were classified as patients with chronic preoperative pain.

### Surgical classes

The patients were divided into 6 surgical classes according to the type of surgery: pharynx surgery, larynx surgery, nasal/ sinus surgery, ear surgery, salivary gland surgery and neck surgery.

### Statistical analysis

The statistical evaluation was performed with R (Version 3.5.0; R Core Team 2018) and IBM SPSS statistics software (IBM SPSS Statistics for Windows, Version 21, Chicago, IL). Pearson Chi-Square test were used to compare categorical variables. The nonparametric Mann–Whitney-U test was used to examine the metric data of two independent groups. One-way analysis of variance (ANOVA) was used to compare more than two groups. To determine the relationship between two metric variables, the nonparametric Spearman correlation was performed. Multivariate linear regression analysis was performed to estimate predictors of postoperative pain including all significant factors from univariate analysis. The significance level was set at *p* < 0.05.

## Results

The patients were 47 ± 17 years old (median age 50 years; range 18–89 years). The population consisted of two third male and one third female patients. The mean CRP level was 15.6 ± 34.6 mg/l (median 2.2 mg/l; range: < 2 to 312 mg/l). The mean LEUK count was 7.8 ± 3.2 Gpt/l (median 7.2 Gpt/l; range: 2.6 to 22.4 Gpt/l).

A correlation analysis between age and CRP showed a positive relationship (*r* = 0.12; *p* = 0.01). No significant correlation could be demonstrated for the number of LEUK (*r* = 0.06; *p* = 0.12).

About one third of patients had a benign or malignant tumor (n = 232). A pairwise multiple comparison showed that patients with peritonsillar abscess had significantly higher values of preoperative LEUK (13.8 ± 3.9 Gpt/l) and CRP concentration (93.0 ± 57.5 mg/l) than other diagnoses (all p < 0.05). The group of patients with a malignant tumor (n = 127) showed significantly higher values of CRP (*p* = 0.03) and the LEUK (*p* = 0.02) compared to other patients (n = 553).

**Fig. 1:**
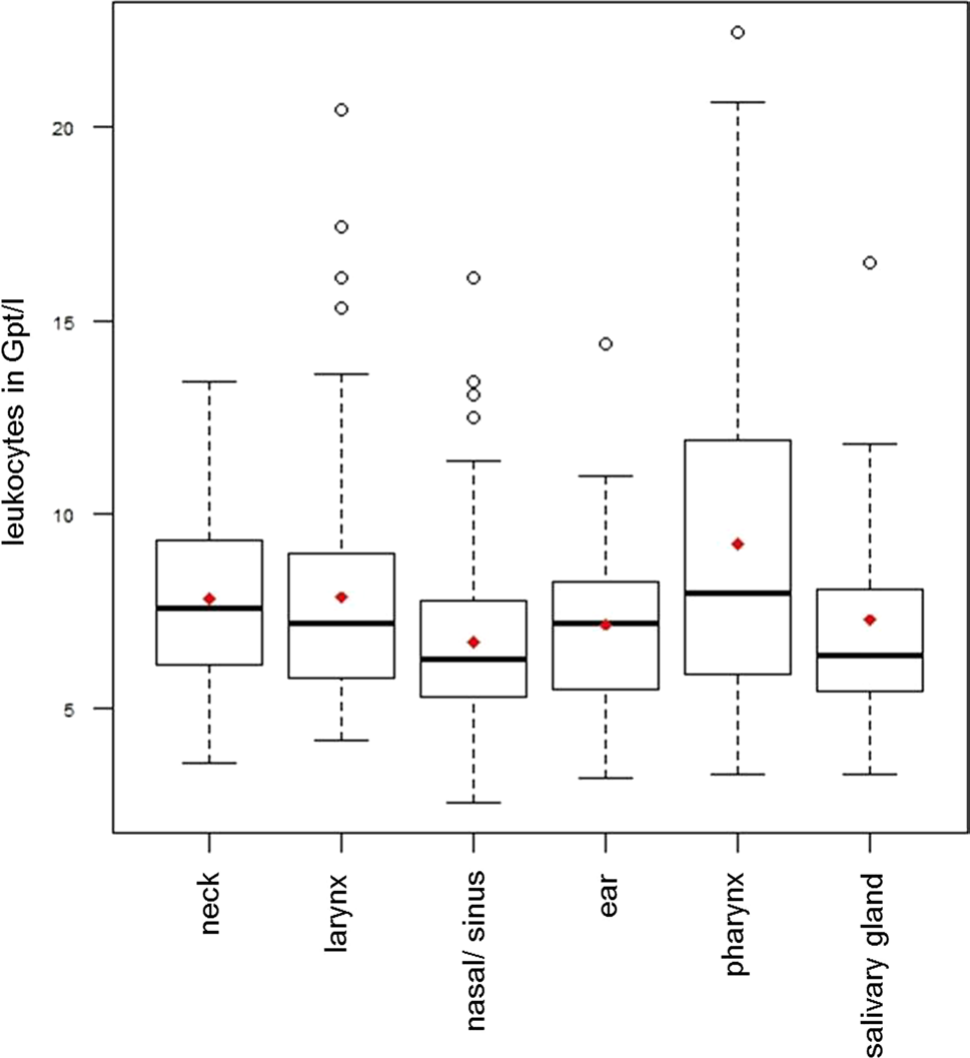
Boxplots showing the preoperative leukocytes values in the different surgical classes (*median* horizontal line, *mean* red rhombus)

**Fig. 2:**
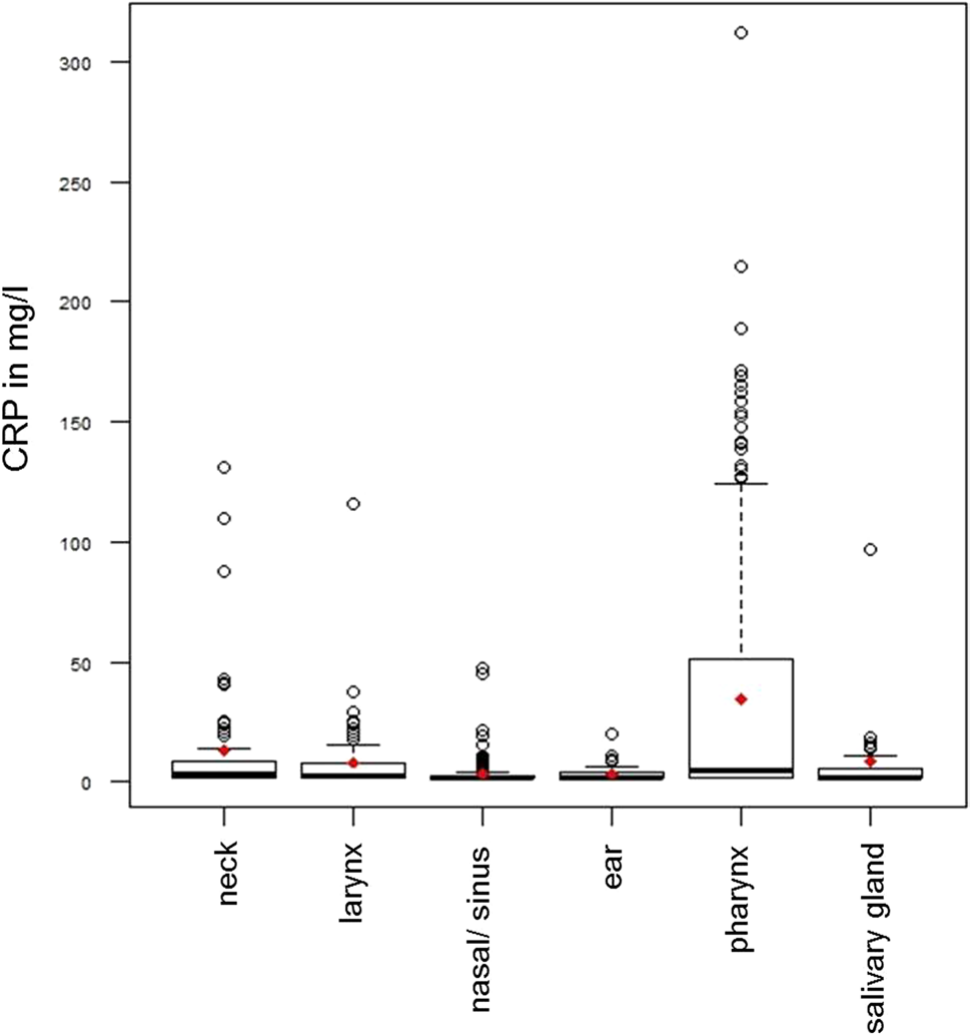
Boxplots showing the preoperative CRP values in the different surgical classes (*median* horizontal line, *mean* red rhombus, *CRP* C-reactive protein)

Patients with pharyngeal surgery had the highest CRP values (34.6 ± 52.9 mg/l) and LEUK concentrations (9.2 ± 4.2 Gpt/l) compared to the other surgical procedures (Figs. 1 and 2). A pairwise multiple comparison with one-way ANOVA showed that the inflammation parameters of this surgical class were significantly higher than those of the others (all *p* < 0.05).

### Perioperative antibiotic therapy

Over half of the patients (*n* = 373) received perioperative antibiotics. Patients in the nasal/ sinus surgery class received antibiotics (*n* = 163), followed by patients in the pharynx group (*n* = 124, *p* = 0.002). There was a significant correlation between the perioperative administration of antibiotics and increased preoperative CRP concentration (*p* = 0.01). No significant relationship between increased leukocyte concentrations and the antibiotic administration could be determined (*p* = 0.19).

### Postoperative pain and inflammatory parameter

**Fig. 3:**
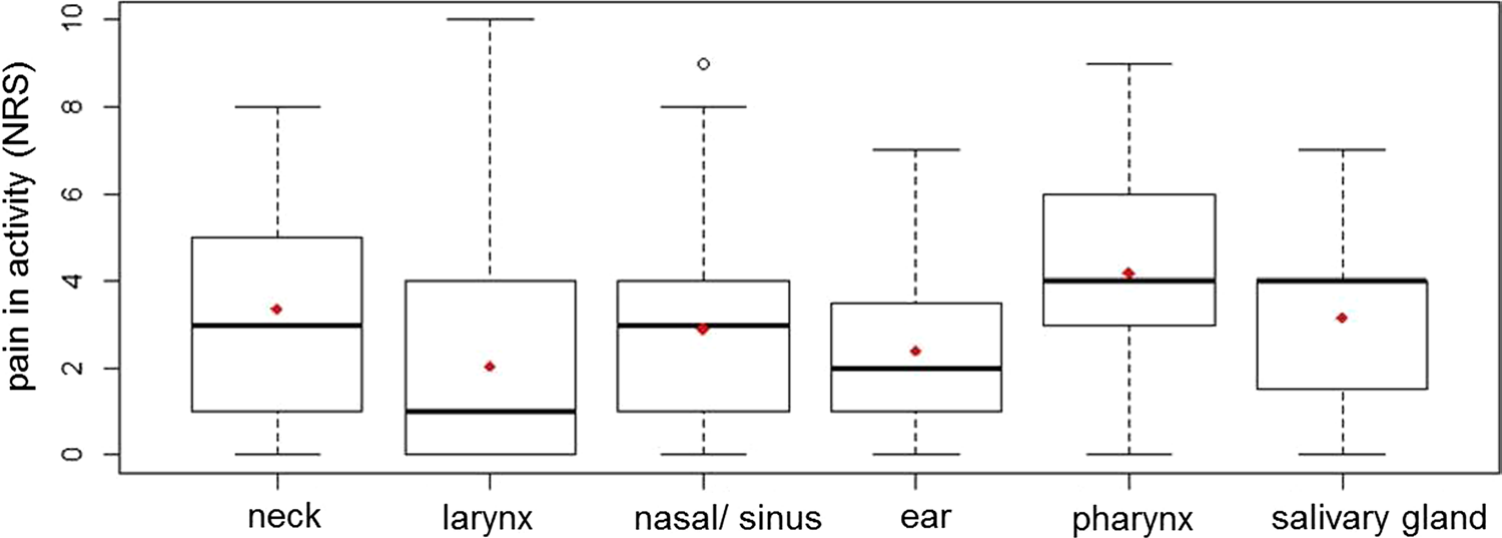
pain in activity at the first postoperative day in different surgical classes (*median* horizontal line, *mean* red rhombus, *NRS* Numeric rating scale

The average pain in activity on the first postoperative day was 3.1 ± 2.4 (NRS). The median NRS was 3.0 (range 0–10). Figure 3 shows pain in activity levels in relation to the various surgical classes. In particular, it shows that patients with pharyngeal surgery reported higher pain compared to patients in other surgical classes. Patients with pharyngeal surgery showed significantly higher pain than patients with larynx, nasal/ sinus or ear surgery (all *p* < 0.05). Patients with neck surgery reported significantly more pain than patients with laryngeal surgery (*p* < 0.05). There was no correlation between postoperative pain and value of CRP (*r* = 0.041, *p* = 0.287, Table 1). Higher pain in activity was associated with LEUK values > 11.3 Gpt/l (*r* = 0.093, *p* = 0.016. Three different multivariate linear regression analysis models were calculated, including or excluding the surgery class or the preoperative chronic pain level. This was done, because surgery class or the preoperative chronic pain level were very dominant factors in the univariate analyses (Table 2). In model 1, without the factors surgery class or the preoperative chronic pain level, the parameters younger age (beta = -0.033; 95% confidence interval [CI] =  − 0.045 to − 0.0203; *p* = 0.0001), female gender (beta = 0.434; CI = 0.065 to 0.804; *p* = 0.021), longer duration of surgery (beta = 0.004; CI = 0.000 to 0.008; *p* = 0.035), and LEUK values > 11.3 Gpt/l (beta = 0.729; CI = 0.174 to1.285; *p* = 0.010) were independently associated with higher postoperative pain. Model 2 without the factor surgery class showed that younger age (beta = -0.035; C] =  − 0.047 to − 0.023; *p* = 0.0001), higher preoperative pain (beta = 0.176; CI = 0.102 to 0.251; *p* = 0.0001), longer duration of surgery (beta = 0.004; CI = 0.000 to 0.008; *p* = 0.037), and LEUK values > 11.3 Gpt/l (beta = 0.644; CI = 0.094 to1.194; *p* = 0.022) were independently associated with higher postoperative pain. There was also a trend to more postoperative pain in female patients (beta = 0.364: CI = − 0.001 to 0.729; *p* = 0.051).

**Table 1:** Correlation analyses between preoperative parameters and postoperative pain

Parameter	Postoperative pain
Age, years	*r* = *− *0.263 **p < 0.0001**
Gender (1 = male; 2 = female)	*r* = 0.099 **p < 0.010**
Charlson comorbidity index (1 = 0; 2 = ≥ 1)	*r* = -0.138 **p < 0.0001**
Preoperative chronic pain (0 = no; 1 = yes)	*r* = 0.127 ***p* = 0.001**
Preoperative chronic pain, NRS	*r* = 0.134 **p < 0.0001**
Duration of surgery, min	*r* = 0.101 ***p* = 0.009**
Perioperative antibiotics (0 = no; 1 = yes)	*r* = 0.052 *p* = 0.173
CRP, (1 = ≤ 7.5 mg/l; 2 = > 7.5 mg/l)	r = 0.041 *p* = 0.287
Leukocytes, (1 = ≤ 11.3 Gpt/l; 2 = > 11.3 Gpt/l)	*r* = 0.093 ***p* = 0.016**

*NRS* Numeric rating scale, *CRP* C-reactive protein; significant values (*p* < 0.05) in bold

**Table 2:** Linear regression analysis for independent predictors of postoperative pain (*R*^2^ = 0.107)

Parameter	beta	Lower 95% CI	Upper 95% CI	Standardized beta	*p*
Model 1 (without the factors surgery class or the preoperative chronic pain level)					
Age, years	− 0.033	− 0.045	− 0.020	− 0.242	**0.0001**
Gender (1 = male; 2 = female)	0.434	0.065	0.804	0.087	**0.021**
Charlson comorbidity index (1 = 0; 2 = ≥ 1)	0.098	−0.349	0.546	0.020	0.666
Duration of surgery, min	0.004	0.000	0.008	0.080	**0.035**
Leukocytes, (1 = ≤ 11.3 Gpt/l; 2 = > 11.3 Gpt/l)	0.729	0.174	1.285	0.097	**0.010**
Model 2 (without the factor surgery class)					
Age, years	− 0.035	− 0.047	− 0.023	− 0.261	**0.0001**
Gender (1 = male; 2 = female)	0.364	−0.001	0.729	0.073	0.051
Charlson comorbidity index (1 = 0; 2 = ≥ 1)	0.075	−0.366	0.516	0.015	0.738
Preoperative chronic pain, NRS	0.176	0.102	0.251	0.173	**0.0001**
Duration of surgery, min	0.004	0.000	0.008	0.078	**0.037**
Leukocytes, (1 = ≤ 11.3 Gpt/l; 2 = > 11.3 Gpt/l)	0.644	0.094	1.194	0.086	**0.022**
Model 3 (without the factor preoperative chronic pain level)					
Age, years	− 0.196	− 0.039	− 0.014	− 0.027	**0.0001**
Gender (1 = male; 2 = female)	0.089	0.068	0.827	0.448	**0.021**
Charlson comorbidity index (1 = 0; 2 = ≥ 1)	0.031	− 0.303	0.615	0.156	0.504
Surgery class (1 = neck; 2 = larynx; 3 = nasal/sinus; 4 = ear; 5 = pharynx; 6 = salivary gland	0.130	0.089	0.367	0.228	**0.001**
Duration of surgery, min	0.108	0.002	0.010	0.006	**0.005**
Leukocytes, (1 = ≤ 11.3 Gpt/l; 2 = > 11.3 Gpt/l)	0.082	0.041	1.173	0.607	**0.036**

Significant values (*p* < 0.05) in bold

*NRS* Numeric rating scale, *CI* confidence interval

Model 3 without the factor preoperative chronic pain level showed again that younger age (beta =  − 0.196; 95% CI = − 0.039 to − 0.014; *p* = 0.0001), female gender (beta = 0.089; CI = 0.068 to 0.827; *p* = 0.021), longer duration of surgery (beta = 0.108; CI = 0.002 to 0.010; *p* = 0.005), and LEUK values > 11.3 Gpt/l (beta = 0.082; CI = 0.041 to 1.173; *p* = 0.036) but also the surgical class (beta = 0.130; CI = 0.089 to 0.367; *p* = 0.000) were independently associated with higher postoperative pain.

### Preoperative chronic pain

About 20% of all patients (*n* = 161) suffered from chronic pain before surgery. About 10% (*n* = 74) of the patients regularly took pain medication before surgery. Most of these were patients with malignant tumors (*n* = 32). The process parameters that were specified by QUIPS are listed in S1 Table. Patients who suffered from chronic pain before surgery reported higher postoperative pain intensity (*p* = 0.001, Table 1). Permanent preoperative intake of pain killers was associated with increased mood impairment and mobility restrictions (*p* = 0.008, *p* = 0.007, Table 3). Furthermore preoperative chronic pain was significant associated with breathing impairments (*p* = 0.03). Higher CRP value was significant correlated with breathing impairments (*p* = 0.01) and night pain (*p* = 0.02). Also, LEUK value was associated with breathing impairments (*p* = 0.0001, Table 3).

**Table 3:** Association of patient characteristics with postoperative pain-related patient-reported outcomes

Parameter	Preoperative chronic pain (1 = yes; 2 = no)	Chronic pain (NRS: 1 = 0; 2 = > 0)	Permanent application of pain killer (1 = yes; 2 = no)	CRP (1 = ≤ 7.5 mg/l; 2 = > 7.5 mg/l)	Leukocytes (1 = ≤ 11.3 Gpt/l; 2 = > 11.3 Gpt/l)
Fatigue	*p* = 0.12	*p* = 1	*p* = 0.26	*p* = 0.14	*p* = 0.31
Nausea	*p* = 0.41	*p* = 0.86	*p* = 0.59	*p* = 0.34	*p* = 0.48
Vomiting	*p* = 0.08	*p* = 1	*p* = 0.73	*p* = 0.11	*p* = 0.27
Mobility impairment	*p* = 0.07	*p* = 1	***p* = 0.007**	*p* = 0.85	*p* = 0.91
Breathing impairment	***p* = 0.03**	*p* = 0.23	*p* = 0.08	***p* = 0.01**	***p* = 0.0001**
Night pain	*p* = 0.14	*p* = 0.93	*p* = 0.35	***p* = 0.02**	*p* = 0.35
Feeling uncomfortable	*p* = 0.05	*p* = 1	***p* = 0.008**	*p* = 1	*p* = 0.33
Desire for more pain killers	*p* = 0.90	*p* = 1	*p* = 0.60	*p* = 0.37	*p* = 0.24

Significant values (*p* < 0.05) in bold

*NRS* Numeric rating scale, *CRP* C-reactive protein

**Fig. 4:**
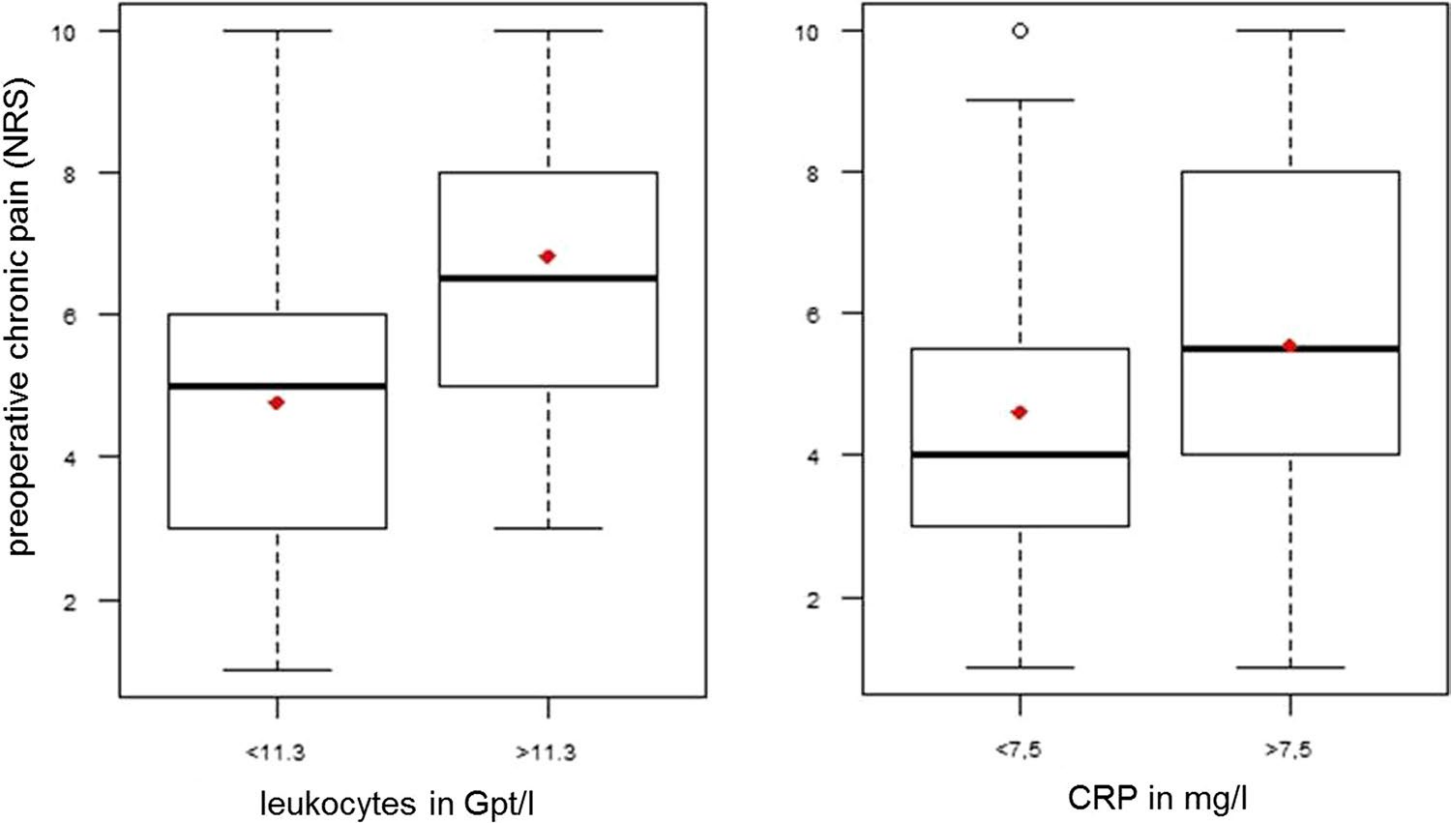
preoperative chronic pain in relation to the preoperative leukocyte and CRP values (*median* horizontal line, *mean* red rhombus, *NRS* Numeric rating scale, *CRP* C-reactive protein)

An ANOVA was performed to determine whether the individual surgical classes differ from each other according to preoperative chronic pain. No significant difference could be found (*p* = 0.46). A Spearman correlation was performed for the total group and the individual surgical classes in order to demonstrate any connections between the preoperative chronic pain and the inflammation parameters. There was a weak positive correlation for the CRP value (*r* = 0.21; *p* = 0.008) as well as for the LEUK level (*r* = 0.19; *p* = 0.01) and more preoperative pain. The results for the individual surgical classes can be found in Table 4. With the exception of the pharyngeal surgery, there were no correlations between inflammation parameters and preoperative chronic pain. Within the pharynx surgery (n = 53), there was a moderate positive linear relationship between CRP, LEUK and preoperative pain. When looking at the subgroup of patients who suffered from chronic pain preoperatively, a significant moderate positive relationship (*r* = 0.49; *p* = 0.02) between CRP and the strength of preoperative chronic pain was shown for the diagnosis of chronic tonsillitis. The Wilcoxon-Mann–Whitney test was also able to show significant differences between the grouped inflammation parameters and the preoperative pain in the total group. Patients with CRP > 7.5 mg/l (*p* = 0.02) and patients with LEUK concentration > 11.3 Gpt/l (*p* = 0.001) had significantly more preoperative pain. Figure 4 shows the differences.

**Table 4:** Preoperative chronic pain and inflammation parameters in different surgical classes

Parameter	Pharynx surgery	Larynx surgery	Nasal/ sinus surgery	Ear surgery	Salivary gland surgery	Neck surgery	All
CRP	*r* = 0.56 **p < 0.0001**	*r* = *− *0.26 *p* = 0.23	*r* = 0.17 *p* = 0.26	*r* = *− *0.40 *p* = 0.22	*r* = 0.58 *p* = 0.16	*r* = 0.21 *p* = 0.43	*r* = 0.21 ***p* = 0.008**
Leukocytes	*r* = 0.53 **p < 0.0001**	*r* = 0.01 *p* = 0.94	*r* = *− *0.02 *p* = 0.88	*r* = 0.00 *p* = 0.98	*r* = *− *0.03 *p* = 0.93	*r* = *− *0.04 *p* = 0.88	*r* = 0.19 ***p* = 0.01**

Significant values (*p* < 0.05) in bold

*CRP* C-reactive protein

## Discussion

The basis for the data collection was the validated QUIPS questionnaire, which was filled out by the patients on the first postoperative day. In the past, QUIPS has proven to be a suitable tool for mapping postoperative pain intensity as well as functional effects and side effects caused by pain therapy [6, 7, 9–17]. One of the strengths of QUIPS is that each survey was conducted uniformly within 24 h of the procedure. The NRS used to measure the pain intensity is sensitive and its validity is well documented [18, 19]. In addition, the pain was only documented on the first postoperative day; i.e. the presented result is primarily reliable only for the first postoperative day. This is a limitation of the study. Nevertheless, typically postoperative pain decreases continuously, i.e. the first postoperative day represents a day with highest postoperative pain [20]. The inflammatory parameters were analyzed only once preoperatively, thus representing only a snapshot of the inflammatory events [21, 22]. Nevertheless, the CRP and LEUK measurement immediately before surgery seems to allow a good prediction of immediate postoperative pain. Hence, a preoperative measurement the day before/at the day of surgery would allow to plan a personalized pain management with the aim to optimally decrease postoperative pain on the first postoperative day. This of course, will also guide the management for the following postoperative days.

In this study, a higher preoperative LEUK value was associated with higher postoperative pain. Because CRP and LEUK represent different responses to acute and chronic inflammation, this could be the reason why CRP was not and LEUK was associated with postoperative pain [23]. In contrast, Kraft et al. showed that CRP but not LEUK is associated with an inflammatory response and wound infection complications in patients after spinal surgery [22]. We could not identify any other study analyzing the effect of preoperative inflammatory markers and postoperative pain.

Beyond LEUK, younger age, duration of surgery, and (as a trend) female gender were other independent predictors of postoperative pain. This confirms results from previous studies [9, 13].

Patients with pharyngeal surgery had the highest CRP values, highest LEUK values and the highest pain in activity levels compared to all other surgical procedures. It is well known that pharyngeal surgery led to the highest pain in otolaryngological surgery [7–9]. Whereas the relation between a peritonsillar abscess (as indication for tonsillectomy/pharyngeal surgery) as a very acute inflammatory disease and increased preoperative inflammation parameters is self-explanatory, it is not self-explanatory in case of tonsillectomy for recurrent acute tonsillitis. In a prior study, the CRP and LEUK values in case of recurrent acute tonsillitis were very normal in most cases compared to the pathologically high values in case of a peritonsillar abscess [24]. Hence, the CRP/LEUK values might be a more reliable parameter to predict postoperative pain than the surgical procedure itself.

When looking at the subgroup of patients who suffered from chronic pain preoperatively, a significant moderate positive relationship between CRP and the strength of preoperative chronic pain was shown for the diagnosis of recurrent acute tonsillitis. CRP can be regarded as potential biomarker for chronic pain [25].

Perioperative antibiotics were not associated with postoperative pain in this study but other authors reported contradictory results. Studies performed within the QUIPS project also received different results. For example, Poller et al. were able to demonstrate a positive effect on postoperative pain and concluded that antibiotic administration prevents local inflammation by bacteria of the oral flora and consequently leads to less nociception [10]. Another study showed that the oral application of honey after a tonsillectomy was associated with decreased pain on the 5th postoperative day, suggesting a similar effect as topical antibiotics on the wound [8]. However, Suffeda et al. came to the opposite conclusion [6] and reported that patients with perioperative antibiotics had more pain here. Since antibiotics are usually administered only in patients with acute or chronic inflammation, the authors hypothesized that the underlying inflammation is the cause of increased postoperative pain. However, the study results listed above are all on the background of a small sample size.

The origin and processing of acute postoperative wound pain is a complex process that is influenced by pro- and anti-inflammatory cytokines [26]. These act rather locally and are only partially reflected by the inflammation parameters in the serum. Possibly, correlations may exist between other inflammation parameters like cytokines and postoperative pain [20, 27]. In further studies, a larger set of inflammatory parameters (for instance like in [24] should be measured preoperatively and on each day postoperatively for 5 to 7 days. Because standard blood tests might have a limited specificity to link inflammation to postoperative pain, metabolomics, i.e. the characterization and quantification of blood metabolites, might allow the detection of more specific genetic, epigenetic, and biopsychosocial alterations results in downstream changes in small molecules linked to postoperative pain [28].

## Conclusion

For the first time it has been shown that the preoperative inflammation parameter LEUK but not CRP was associated with pain on the first postoperative day. Further studies are required to verify these results and develop standardized pain protocols for patients with high preoperative LEUK value.

## Supplementary Information

Below is the link to the electronic supplementary material.

Supplementary file1 (DOCX 27 KB)

## Data Availability

All data of this study are available on request from the corresponding author.
