# Supplementary material for: Association between preoperative serum C-reactive protein level and leukocyte count and postoperative pain after otolaryngological surgery
Source: Eur Arch Otorhinolaryngol. 2023 Apr 20;280(8):3877–84. doi: 10.1007/s00405-023-07980-4 (PMC10313535; doi:10.1007/s00405-023-07980-4)
Supplement: Supplementary file 1 — Supplementary file1 (DOCX 27 KB) [file 405_2023_7980_MOESM1_ESM.docx]

**Supplement Table S1 Table**

| **Supplement Table S1 Table:** QUIPS process parameter. | | |
| --- | --- | --- |
| **Parameter** | **n** | **%** |
| Local anesthesia in operating area  no  yes  missing | 202  252  226 | 29.7  37.0  33.2 |
| Premedication  no  yes  midazolam  clonidine  tranxilium | 44  622  610  9  2 | 6.6  93.3 |
| Intraoperatively remifentanil  no  yes | 113  563 | 16.6  82.7 |
| Intraoperatively clonidine  no  yes | 636  40 | 93.5  5.8 |
| Intraoperatively ketamine  no  yes | 673  3 | 98.9  0.4 |
| In recovery-room non-opioid  no  yes  metamizole  acetaminophen  ibuprofen  parecoxib | 450  229  154  72  2  1 | 66.2  33.7 |
| In recovery-room opioid  no  yes  piritramid  pethidine  clonidine  tramadol | 510  169  163  3  2  1 | 75.1  24.9 |
| On ward non-opioid  no  yes  metamizole  acetaminophen  ibuprofen  etoricoxib | 251  429  231  36  105  54 | 36.9  63.0 |
| On ward opioid  no  yes  piritramid  tilidin  clonidine  tramadol  oxycodone  fentanyl  buprenorphine  tapentadol | 543  136  98  5  1  26  1  2  1  2 | 79.8  20.0 |
| Pain documentation  no  yes | 168  512 | 24.7  75.2 |
| Education about pain therapy  no  yes, even special  yes, just general | 56  188  436 | 8.2  27.7  64.3 |
